# Epigenetic Landmarks of Leaf Senescence and Crop Improvement

**DOI:** 10.3390/ijms21145125

**Published:** 2020-07-20

**Authors:** Agnieszka Ostrowska-Mazurek, Piotr Kasprzak, Szymon Kubala, Magdalena Zaborowska, Ewa Sobieszczuk-Nowicka

**Affiliations:** 1 Department of Plant Physiology, Faculty of Biology, Adam Mickiewicz University, ul. Uniwersytetu Poznańskiego 6, 61-614 Poznań, Poland; agnost2@amu.edu.pl (A.O.-M.); piokas2@st.amu.edu.pl (P.K.); 2 Institute of Biochemistry and Biophysics, Polish Academy of Sciences, ul. Pawińskiego 5A, 02-106 Warsaw, Poland; szymon.globus@ibb.waw.pl (S.K.); magdalena.zaborowska@gmail.com (M.Z.)

**Keywords:** leaf senescence, epigenetic regulation, histone modifications, DNA methylation, chromatin remodeling, stress response, crop improvement

## Abstract

This review synthesizes knowledge on epigenetic regulation of leaf senescence and discusses the possibility of using this knowledge to improve crop quality. This control level is implemented by different but interacting epigenetic mechanisms, including DNA methylation, covalent histone modifications, and non-covalent chromatin remodeling. The genetic and epigenetic changes may act alone or together and regulate the gene expression, which may result in heritable (stress memory) changes and may lead to crop survival. In the review, the question also arises whether the mitotically stable epigenetic information can be used for crop improvement. The barley crop model for early and late events of dark-induced leaf senescence (DILS), where the point of no return was defined, revealed differences in DNA and RNA modifications active in DILS compared to developmental leaf senescence. This suggests the possibility of a yet-to-be-discovered epigenetic-based switch between cell survival and cell death. Conclusions from the analyzed research contributed to the hypothesis that chromatin-remodeling mechanisms play a role in the control of induced leaf senescence. Understanding this mechanism in crops might provide a tool for further exploitation toward sustainable agriculture: so-called epibreeding.

## 1. Introduction

In eukaryotic cell nuclei, genomic DNA is packaged into a highly organized nucleoprotein complex known as chromatin. The fundamental unit of chromatin is the nucleosome, which is composed of DNA wrapped around a core of eight histone molecules. Nucleosomes are not simply static structural units. Nucleosomes can be moved, stabilized/destabilized, and disassembled/reassembled at particular genome locations in response to specific environmental signals or developmental cues. The resulting dynamics of the chromatin structure directly modulate the DNA accessibility, thus regulating all DNA template processes (i.e., transcription, DNA replication, DNA repair, recombination, transposition, and chromosome segregation) and affecting various processes in plants such as root growth, flowering timing, floral organogenesis, gametophyte or embryo formation, senescence, as well as the response to pathogens or environmental changes [1,2]. However, not all genes are active at all times. Therefore, cells use several mechanisms to alter the chromatin structure and the properties of a nucleosome to specifically control gene expression. Regulation of gene expression within the chromatin context is controlled by different mechanisms, including nucleosome assembly, ATP-dependent nucleosome reorganization, DNA methylation, and post-translational covalent histone modifications (e.g., acetylation, ubiquitination, methylation, phosphorylation, sumoylation). Different epigenetic regulators control all the above mechanisms, and the changes in these regulators can influence gene expression of a particular gene or set of genes, while the underlying DNA sequence remains identical [3,4].

In the last two decades, researchers have accumulated a wealth of knowledge providing evidence of the prevalence of epigenetic variability (natural as well as generated) and its potential to influence the phenotype (agronomic traits) and bring large crop improvements. Can the mitotically stable epigenetic information be used for crop improvement? The answer is very similar to that for transcriptional factors. Chromatin changes also control plant morphology and response to the environment, and greater control over traits may be achieved by understanding these mechanisms, which is highly important from the breeding point of view [5]. There are several cases of naturally occurring epialleles (i.e., DNA methylation alleles), that are independent of DNA sequence variation, causing a visible phenotype. Dynamic DNA methylation is believed to target two main functions: firstly, regulation of gene expression by methylation and demethylation at gene promoter and/or body sites; and secondly, protection of genome stability by silencing of repeat sequences, such as transposable elements (TEs), described in *Linaria vulgaris* [6], *Solanum lycopersicum* [7], *Sinapis alba* [8] and *Oryza sativa* [9].

This review summarizes and synthesizes knowledge on epigenetic regulation of leaf senescence and discusses the possibility of using this knowledge to improve crop quality. It is all based on empirical data, among others from transgenic analysis of mutants of *Arabidopsis* and cereal lines with defects within the genes involved in DNA methylation, modification of histones, and ATP-dependent reorganization of nucleosomes.

## 2. Epigenetic Landmarks and Plant Senescence Biology

### 2.1. Stress-Induced Versus Developmental Senescence Model

Senescence is a ubiquitous phenomenon in the biological world. From an ontogenetic perspective, senescence is now established as a developmental and genetic program acquired during evolution [10]. In plants, senescence is a prelude to cell (organ) death, and during this process metabolites and macromolecules released are reutilized for plant growth. Generally, senescence occurs before programmed cell death (PCD), since symptomatic leaf yellowing can be reversed based on the timing of senescence, while PCD is a terminal, irreversible program. It has been suggested that the term “programmed cell death” in plants should be restricted to the specific stage of the intrinsic senescence program when it has reached a “point of no return” and leaf yellowing is no longer reversible [11]. In the dark-induced leaf senescence model (DILS) we have also shown the role of autophagy in the metabolic turnover of cell components as one of the quality control mechanisms of barley-induced leaf senescence, which is crucial for the efficient performance of the process [11].

Studies on *Arabidopsis* as a model organism for leaf senescence have revealed several facets of senescence. A theoretical model has been put forward about how the capacity for senescence is formulated during leaf development and possibly how internal and external factors are integrated with age to define the timing of senescence [12]. In *Arabidopsis,* leaves have a very short lifetime and senescence seems to start as soon as full expansion is reached. Moreover, the senescence of the individual rosette leaves may not be closely linked to the developmental stage of the plant. In many plants, such as the pea, removal of the developing flowers and pods will significantly extend the life of the leaves, but in *Arabidopsis*, male-sterile mutants, or plants from which the developing bolts were removed, did not show any extension to the lifetime of the individual leaves [13,14,15].

In cereals, senescence appears to be regulated at the level of the individual leaf. Nutrients are thus mobilized from the older leaves to the younger leaves and eventually to the flag leaf, which contributes the majority of the nutrients and photoassimilates used for charging of the grain [16].

The study of leaf senescence has been complicated by the lack of coordinated development of the cells within an individual leaf, and various methods have been used to artificially induce senescence to obtain a synchronous process. For example, dark-induced senescence has been used frequently as a useful method to induce synchronous senescence as many typical senescence symptoms such as chlorophyll degradation and loss of protein occur [11,17,18,19,20]. Sobieszczuk-Nowicka et al. [11] reported evident differences in gene medleys between dark-induced leaf senesce (DILS) and developmental senescence. Also, Law et al. [17] propose a model illustrating the specific metabolic strategies employed by leaves in response to two darkening treatments in *Arabidopsis*: an individually darkened leaf, which supports rapid senescence, and a leaf from a whole darkened plant, characterized by a strong capacity for survival. Several external stimuli, other than darkness, can also induce the onset and progression of senescence, or processes resembling senescence and sharing some common pathways. These include environmental stresses such as extremes of temperature, water stress, nutrient deficiencies, wounding and light conditions, as well as pathogen attack and the hypersensitive response.

Plants cannot move away from adverse environmental conditions, and senescence is a mechanism that they have evolved to cope with a problem. In these cases, recycling of nutrients is likely an important concern wherever possible. Senescence refers to the process or condition of growing old (from Latin senescere, to grow old). The current physiological understanding of a state of senescence supports a definition that considers senescence (i) to be the developmental phase that is an episode of transient differentiation at the end of growth, (ii) may or may not be replaced by death, and (iii) is entirely dependent on cell viability and expression of specific genes. Green tissues may also pass directly from maturity to death without a clear intervening senescence phase. Such genetic interventions show that death neither requires senescence nor is the inevitable consequence of it [10].

What is more, it is extremely difficult to uncouple senescence regulatory pathways from stress responses, since the genetic program(s) underlying senescence largely overlaps with that of plant defense. Likewise, many senescence-associated genes (SAGs) involved in developmental senescence have also been found to have a role in other biological processes, and gene mutations involved in diverse facets of plant growth and development can alter leaf senescence. This indicates that there is an overlap between the senescence network and other biological networks, or that genes that have evolved for one purpose have been recruited for other purposes [21]. Becker and Apel [22] incubated barley leaf segments in the dark and found three mRNAs that increased in level. Two of these were also induced by wounding, drought stress, jasmonate, and abscisic acid (ABA) and repressed by cytokinin, but were not detectable in naturally senescing leaves, indicating that they may be part of the stress response, rather than senescence. The epigenetic variability may also be considered as a good marker to differentiate these two processes.

### 2.2. Epigenetic Mechanisms in Plants

In plants, the cell-specific gene expression scheme determined by the state of chromatin is crucial to establish and maintain the functionality of a particular cell or group of cells, which clearly indicates the presence of various epigenomes differing in the pattern of post-translational modifications (PTMs) conditioned by epigenetic marks [23]. All these changes influencing gene expression are the basis for proper regulation of biological processes in response to the environment. The specificity of functioning of these mechanisms throughout the genome promotes the accumulation of marks causing or maintaining the same state, which aims to ensure cell/organism stability [24,25].

The specific characteristics of the modifications, referred to as the histone code, together with the methylation patterns, condition the plasticity of the complex chromatin structure. Type and location of various PTMs of the histones N-terminal lysine, serine, threonine, and arginine residues determine the availability of the DNA sequence for the transcription factors, thereby controlling the expression of individual genes [26,27]. The primary mechanism of epigenetic histone regulation includes acetylation of lysine residues mediated by histone acetyltransferases (HATs), enhancing gene expression, and deacetylation with histone deacetylases (HDACs), conditioning gene activity silencing [28,29]. The antagonistic relation between HATs and HDACs helps to maintain the homeostatic balance [30]. Histone methylation also participates in the formation of the chromatin structure, occurring on lysine or arginine residues. The level of activation/deactivation depends not only on the site affected, but also on the number of methyl groups added [31].

Cytosines in DNA of eukaryotes may undergo methylation mediated by DNA methyltransferases (DNMT). The high level of 5-methylcytosine is specific for the inactive state of chromatin. This mechanism is essential for silencing repetitive sequences and transposable elements (TEs), DNA sequences which can change their position within a genome, to avoid uncontrolled transposition, which allows for genome integrity maintenance [20]. In plants, it can be categorized into three types: symmetric CG or CHG (H is A, T or C) and asymmetric CHH DNA-methylation. It acts differently in genes, where DNA methylation is restricted to CG sites. The presence of methyl groups within the gene promoter in most cases leads to down-regulation or silencing expression, while the effect of DNA methylation appearing in the gene body remains unclear [32,33]. Methylation at the 5′ position of cytosine is considered as one of the most common mechanisms of epigenetic regulation [34,35].

ATP-dependent chromatin remodeling plays a key role in regulating the accessibility of individual DNA regions, which must be released from the nucleosome tangles to undergo transcription. Chromatin can be changed not only by covalent DNA and histone modifications [36]. The basis for the functioning of active remodeling is the use of energy from ATP hydrolysis to modulate the structure of nucleoprotein, which directly affects the expression of selected genes [37]. Numerous studies have proven that ATP-dependent chromatin remodeling factors play an important role in regulating plant response to stress factors. Three classes of ATP-dependent chromatin complexes have been distinguished: the SWI/SNF ATPases, the imitation switch (ISWI) ATPases, and the chromodomain and helicase-like domain (CHD) ATPases. Although each class appears to be associated with the regulation of a specific group of plant responses, details of functioning interdependency are not yet known [38].

### 2.3. Epigenetics and Leaf Senescence

Leaf senescence, both developmental and stress induced, as mentioned above, is controlled by a multilevel regulatory network, and the dynamics of cooperation among all signal pathways is conditioned by plant phenotypic plasticity under different conditions [5,34]. Epigenetic mechanisms constitute another regulatory layer affecting the activity of transcription factors (TFs) controlling the process and inducing the functionality of SAGs as the next stages of development proceed. The diversity of histone modification types determines the complexity of their functions, just as their type depends on the range of activity [31,34]. The association between histone modification and senescence-related gene expression has been described mainly for H3 and H4 histones. Transcription changes are observed as activating (e.g., H3K4me2/me3—histone H3 at lysine 4 di-/trimethylation) and repressing (e.g., H3K27me2/me3—histone H3 at lysine 27 di-/trimethylation) marks, and they lead to differential gene expression, resulting in altered metabolism. The effect of the epigenetic landmark also depends on its accumulation and position on the gene. Regulation of the senescence process by methylation is supported by the variable level of expression of individual methylation enzymes, proteins conducting cytosine methylation/demethylation, such as MET1 (DNA methyltransferase1), CMT3 (chromomethylase3), ROS1 (repressor of gene silencing 1), DME (DEMETER protein—a potential transcriptional activator that may act by nicking the target promoter; it catalyzes the release of 5-methylcytosine (5-meC) from DNA by a glycosylase/lyase mechanism), and DML2/3 (DEMETER-like proteins), but the specific mechanism is still not described. However, the involvement of DNA methylation in maintaining genome stability by silencing TEs and repetitive sequences during the senescence progress has been confirmed by research [39,40]. DNA methylation localized at the promoter and histone H3 at lysine 9 dimethylation (H3K9me2) are considered to be heterochromatic marks, which inhibit gene expression [41,42]. Despite obvious conclusions, there is still no clear evidence of a direct relationship in which the accumulation of methylation marks contributes to the progression of senescence. Therefore, it is worth exploring whether DNA methylation can be considered as a cause, an effect, or whether the answer to this question is equivocal. Chromatin structure changes are a natural consequence of histone modification, DNA methylation and ATP-dependent remodeling, and they play a key role in regulating the accessibility of individual DNA regions [36,43]. Up-regulation of leaf senescence by members of the SWI/SNF family remodelers still lacks a description of the mechanism, while AT-hook protein ORESARA 7 (ORE7) is considered to repress chromatin decondensation, blocking access to leaf senescence transcription factors.

There follows a detailed description of changes conditioning the progression of leaf senescence processes that occur under the influence of post-translational histone modifications, DNA methylation, ATP-dependent chromatin remodeling and additional epigenetic regulation mechanisms. Examples of loss-of-function and gain-of-function *Arabidopsis* mutants and transgenic lines showing senescence-affected phenotype are summarized in Table 1.

#### 2.3.1. Leaf Senescence-Based Histone Modifications

Leaf senescence initiation conditioned by both cellular signaling and global regulation of transcription is aimed at reducing the activity of genes involved in photosynthesis, while SAGs are sequentially turned on. The complexity of the process requires dynamic epigenetic regulation of chromatin plasticity including histone modifications and the use of these variants in ATP-dependent chromatin remodeling [58]. Although the precise mechanism of target recognition in senescence regulation in most cases remains unknown, the importance of the functions of enzymes in regulating this process has been confirmed in numerous studies based on transcriptomic analysis of mutants and transgenic lines.

The global impact of changes in the histone deacetylation profile on the regulation of senescence has been shown in studies on transgenic *Arabidopsis* plants with expression of the antisense histone deacetylase 1 (AtHD1) gene, or in HDA6 axe1-5 mutant and histone deacetylase 6-RNA-interfering mutant (HDA6-RNAi), both resulting in accumulation of acetylated histones which occurred together with changes in senescence timing in different manners [44,45]. Loss of HDA6 activity most likely leads to senescence-specific cysteine protease (SAG12) and xyloglucan endotransglucosylase/hydrolase (SEN4) expression downregulation and senescence delay, while histone deacetylase 9 (HDA9) induces senescence, which can be supported by the fact that HDA9 mutants show postponed yellowing and leaf senescence [46,59].

Enhancing gene expression by direct histone acetylation was initially shown in the example of acetyltransferase Elongator. Silencing of the gene for acetyltransferase Elongator was correlated with the reduction of small Rubisco subunit (ribulose-1,5-bisphosphate carboxylase/oxygenase) gene transcription and it led to acceleration of tomato leaf senescence [60].

Another well-studied histone modification involved in leaf senescence is acetylation of Lys-9 of H3 histone (H3K9ac). A study based on chromatin immunoprecipitation (ChIP) and RNA analysis showed that H3K9ac marks many upregulated genes during senescence, mainly activating TFs, such as WRKY53, at different phases of leaf development. H3K9ac is similar in nature to H3K4me3. Although the average number of H3K9ac marks increases around genes associated with leaf senescence (such as WRKY53 and probable WRKY transcription factor 41—WRKY41), it is much smaller than that of H3K4me3. The H3K9ac levels are high during the early stages of senescence and decrease toward the end of it. This proportion is contrary to that of H3K4me3 levels, as at first they are below average and increase by a great margin as senescence progresses [61]. Transgenic barley with RNAi silenced single-stranded DNA-binding protein WHY1 (HvWHIRLY1), a major organizer of chloroplast nucleoids, shows delayed leaf senescence in intensive light and drought stress conditions, which suggests its upstream role in the plants’ response to stress-induced senescence. These plants under drought stress showed no increase in acetylation (H3K9ac) levels in promoter and coding regions of the senescence-associated genes, especially HvS40, encoding a putative regulator of leaf senescence, indicating the epigenetic nature of process regulation [62,63].

Histone methylation may certainly have an equally strong influence on regulation of leaf senescence as acetylation. The presence of a methyl group/groups on lysine or arginine residues promotes the attachment of regulatory proteins and increases the transcriptional availability of DNA, loosening the structure of the nucleosome [58].

Most cases of methylation in *Arabidopsis* are related to H3 histone. Histone 3 lysine 4 trimethylation (H3K4me3) has been described as a distinct landmark of leaf senescence. During this process, H3K4me3 significantly increases, mostly within WRKY53, a key regulator of early stages of senescence, regulating the activity of many SAGs and TFs during senescence [64,65]. Studies have shown that H3K4me3 appeared in greater numbers for DNA regions of *Arabidopsis thaliana* plants that were growing under dark-induced stress, rather than in plants under normal light conditions, mainly for exon regions. Chromatin immunoprecipitation-sequencing (ChIP-seq) analysis proved that H3K4me3 changes could be observed in dark-induced leaf senescence, as well as during aging. H3K4me3 is proven to be associated predominantly with active genes. Although H3K4me3 has been demonstrated to occur in leaf senescence, some genes with high levels of expression in senescent leaf tissue lacked H3K4me3 marks [66,67]. According to one of the proposed mechanisms of H3K4me3 functionality, JMJ16, an *Arabidopsis* JmjC-domain-containing protein which may act as histone H3 lysine demethylase, delays leaf senescence through repression of WRKY53. Decrease of JMJ16, the specific H3K4 demethylase, is correlated with the increase of H3K4me3 as senescence progresses [49].

Histone 3 lysine 27 trimethylation (H3K27me3) is considered to be another epigenetic marker of leaf senescence. The number of these marks decreases during senescence, as this methylation prohibits overexpression of key regulatory and functional genes mostly involved in the regulation of developmental leaf senescence. This is caused by genes such as REF6, a plant-unique H3K27 demethylase, which functions as a putative binding protein for the promoter of NYE1 (NONYELLOWING1), the gene encoding senescence-inducible chloroplast stay-green protein, a regulator of chlorophyll (Chl) degradation. REF6 promotes Chl degradation during leaf senescence by up-regulation of NYE1 [50,68].

Leaf senescence involves global alterations in chromatin organization including H3K4me2, H3K4me3, H3K27me2, and H3K27me3 modifications designating marks associated with regions’ activation or inactivation, respectively. Ay et al. [51] initially studied the redistribution of histone methylation marks at the WRKY53 senescence regulator. A significant increase of H3K4me2 and H3K4me3 was observed at the 5′ end and coding regions connected with activation of this locus during senescence. On the other hand, plants with overactive SU(VAR)3–9 HOMOLOG 2 (SUVH2) histone methyltransferase, a key gene silencer acting by maintenance of a compact chromatin structure, exhibited delayed leaf senescence. It was caused by inhibition of key senescence regulators, such as SIRK (senescence-induced receptor-like serine/threonine-protein kinase) or SAG101 (senescence-associated carboxylesterase 101), related to repression of WRKY53 function. Increased levels of H3K27me2 and H3K27me3 were then observed at the 5′-end region of WRKY53 [51,69]. Further large-scale transcriptomic analysis showed that overexpression of SUVH2 histone methyltransferase limits the activity of nearly half of the senescence-related regulatory factors (SRRFs) [52]. The latest research has demonstrated that SUVH2 also operates as a downstream element of ATM (ataxia telangiectasia mutated) encoding serine/threonine-protein kinase, which is the major transducer of the double-strand break (DSB) signal, in DSB-induced leaf senescence of *Arabidopsis*. ATM activity leads to repression of DSB-dependent transcription of SAGs, including WRKY and NAC TFs (one of the largest families of transcription factors in plants). This path of regulation is based on histone lysine methylation. The presented data suggest that accumulation of DSBs may lead to SAG activation connected with modulation of H3K4me3/H3K27me3 marks manifested by accelerated senescence [53,70].

#### 2.3.2. DNA Methylation Changes

DNA methylation plays an important role as an epigenetic mark and greatly influences plant development, by changing chromatin structure in a number of ways. The results of many studies confirm that dynamic methylation changes during plant senescence, in contrast to the global range of chromatin adjustments, are local. In *Arabidopsis* methylation is mediated by three enzyme families: methyltransferases (METs), the DOMAINS REARRANGED METHYLTRANSFERASES (DRMs), and the plant specific CHROMOMETHYLASES (CMTs). Research on a transgenic line with an antisense MET1 gene confirmed the relevance of enzyme activity, where delayed senescence and other developmental defects were observed [54]. Moreover, the study carried out by Ogneva et al. [71] suggested that age-associated changes in DNA methylation levels are related changes in the activity of methylation/demethylation enzymes. This was supported by the *Arabidopsis* plant transcript analysis, which showed a clear decrease in expression of the methyltransferase genes AtCMT3 and AtMETI, while the levels of the demethylase genes AtROS1, AtDME, AtDML2 and AtDML3 rose at least at some phases of plant senescence [71]. There is also a clear connection between DNA methylation change and the levels of TEs. It has been found that TEs are released in *Arabidopsis* and barley during senescence [72,73]. TEs are potentially mutagenic when active and there may be a correlation between TE activation/inactivation and effects on the stress response [40]. This TE activation during senescence can, as the research has shown, affect the expression of neighboring genes, although this process should be investigated in the future in more detail [73]. A recent study suggested that DNA methylation of the retrotransposon NMR19-4 (naturally occurring DNA methylation variation region 19) correlates with changes in this epiallele’s control over leaf senescence. The study classified NMR19 as NMR19-4 and NMR19-16 based on its location. It was discovered that DNA methylation of NMR19-4 negatively regulates the expression of pheophytin pheophorbide hydrolase (PPH), which is an enzyme involved in chlorophyll breakdown during leaf senescence. It was concluded that DNA methylation of the NMR19-4 epiallele can be a regulatory factor of this process in *Arabidopsis thaliana*. Nevertheless, it is not the sole factor regulating PPH expression, since complex regulatory mechanisms are involved in leaf senescence [74,75,76].

The latest study conducted by Trejo-Arellano et al. [20] showed significant downregulation of methylation pathway elements responsible for maintaining the integrity of the chromatin during dark-induced senescence, e.g., RdDM (RNA-directed DNA methylation) and DDM1/CMT2 (nucleosome remodelers: decreased DNA methylation 1/CHH methyltransferase) correlated with CHH methylation and heterochromatin at chromocenter decondensation. However, only local changes in methylome were present. A similar observation was made earlier in developmental senescence [51]. The examples presented support the dynamics of DNA methylation during plants’ final phase of development and highlight the significance of this regulatory mechanism in epigenetic reprogramming for plant life.

#### 2.3.3. Chromatin Remodeling Adjustments

Chromatin structure can be changed not only by covalent DNA and histone modifications [36]. There is a special group of remodelers that use energy for nucleosome repositioning and control gene expression by revealing the sequence for transcription machinery [43]. Such complexes, as mentioned, use energy derived from ATP hydrolysis to change the chromatin structure. Chromatin remodeling complexes may move nucleosomes along the same strand (*cis*) as well as between different threads (*trans*) [73]. Moreover, they can remove nucleosomes from the regulatory DNA regions [74], change the nucleosome structure without changing its position on the DNA strand [75] or loosen the nucleosome structure [76]. Among such remodelers that use energy derived from ATP are the SWI/SNF complexes. The core of the *Arabidopsis* SWI/SNF complex is composed of: one of four SWI2/SNF2-type ATPases (BRM, SYD, CHR12 and CHR23); two of four SWI3-type proteins (SWI3A, SWI3B, SWI3C and SWI3D) and one SNF5-type protein (BSH) [77,78]. Investigating in more detail data reported by Li et al. [79] (supplementary materials), we found that REF6 facilitates the recruitment of BRM. BRM directly targets many genes that are involved in leaf senescence: *SAG21* (SAG21/AtLEA5 belongs to the late embryogenesis-, root development and stress response-associated redox-related LEA protein family); *SAG101* (senescence-associated carboxylesterase); *NAC6* (NAC-domain transcription factor 6); *SAUL1* (senescence-associated e3 ubiquitin ligase 1); *SFP1* (sugar-porter family protein induced during leaf senescence); *AOC1*; *AOC2*; *AOC3* (allene oxide cyclase 1,2,3). Moreover, BRM can colocalize senescence-dependent genes, for example SAG21 or SFP1, with REF6. These supplementary data suggests that BRM containing SWI/SNF complexes can be involved in the nucleosome control of senescence-dependent genes in plants. Moreover, they suggest that BRM-containing SWI/SNF complexes may cooperate with REF6 during epigenetic control of leaf senescence.

Chromatin remodeling protein 1 (DRD1), whose epigenetic function has been described as a gene silencer from the SWI2/SNF2 subfamily, was also identified [55] as a leaf senescence regulator. Drd1-6 mutants, overexpressing the DRD1 gene, show significant SAG inhibition during dark-induced and natural senescence and delayed process progression. A similar effect was obtained earlier by overexpressing another chromatin remodeler, an AT-hook DNA binding protein ORE7, which specifically binds AT-rich DNA sequences, blocking access for other TFs. The intensity of observed transcription regulation was dose-dependent [56,80]. DDM1 (ATP-dependent DNA helicase DDM1), belonging to the same group of proteins as DRD1 and ddm1-2 mutants, is also responsible for the occurrence of mutations in the helicase domain. A Ddm1-2 versus wild type test confirmed the role of the helicase superfamily C-terminal (HELICc) domain of SWI2/SNF2 chromatin remodelers in regulating the leaf senescence process [55].

#### 2.3.4. Other Epigenetic Mechanism Modifications

The influence of microRNAs (miRs) on leaf senescence regulation has been demonstrated in numerous studies to date. It is worth noting that miRNA expression can be spatially dynamic and specific, which determines the diversity of their functions. The nature of the plant’s response also plays a significant role here [81]. Multilevel functionality of molecules such as miR164, miR319, and miR396 was recognized as positive senescence regulator through expression inhibition (miR164—ORE1, NAC transcription factor family member), repression of signaling pathway members (miR319—DNA-binding transcription factor) and local limitation of regulators activity (miR396—growth-regulating factor, GRF) [82,83,84]. Delayed leaf senescence phenotype was also observed in *Arabidopsis arf2* mutants. Auxin response factor 2 (ARF2), which binds to the DNA 5’-TGTCTC-3’ sequence, found in the auxin-responsive promoter elements, is responsible for auxin-dependent senescence progression, and it can be knocked out by miR390-mediated production of TAS3—transcriptional silencing complex protein [57,85].

The latest research results reveal the circadian evening complex as a mediator of *Arabidopsis* leaf senescence regulation. The main function of the evening complex is to maintain the correct operation of the circadian clock, especially by affecting its gene expression. It also acts as an environmental sensor, conveying information about temperature and light entrainment to growth and developmental pathways [86]. It was revealed that this complex can repress *Arabidopsis* jasmonate-induced leaf senescence, by binding the promoter of transcription factor MYC2, which encodes a key activator of this signaling pathway, and represses its activation. The study of physiological phenotypes, as well as the measurement of expression level of jasmonate-induced SAGs, showed that the leaf senescence was significantly upregulated in evening complex mutants [87]. Other research confirms that the circadian clock can also positively regulate leaf senescence through the pseudo-response regulator 9 (PRR9), its morning complex component. PRR9 controls the ORE1 regulator, repressing miR164 by binding directly to this promoter. Through genetic analysis of the *prr9* mutant, a research team proved that ORE1 overexpression eliminates mutants’ delayed age-induced leaf senescence [88].

A different type of epigenetic regulation was observed during a study on the impact of ATM kinase on leaf senescence triggered by DNA DSBs [82]. It was found that ATM suppresses leaf senescence in *Arabidopsis* through epigenetic control of SAGs. By examining the senescence phenotypes in the loss-of-function mutants for 13 key components of the DNA repair pathway, it was confirmed that the deficiency in ATM results in premature senescence in *Arabidopsis*. The epigenetic mechanism of this modification relies on ATM repressing the expression of senescence-associated TFs such as ANAC016 (NAC domain-containing protein 16; it promotes leaf senescence by up-regulating SAGs in response to stress-induced and developmental senescence signals), WRKY6 (WRKY transcription factor 6), WRKY53 and WRKY75 (probable WRKY transcription factor), through modulating histone lysine methylation [53]. 

## 3. PERSPECTIVES: Exploiting Epigenetic Regulation of Senescence in Crop Improvement

**Figure 1:**
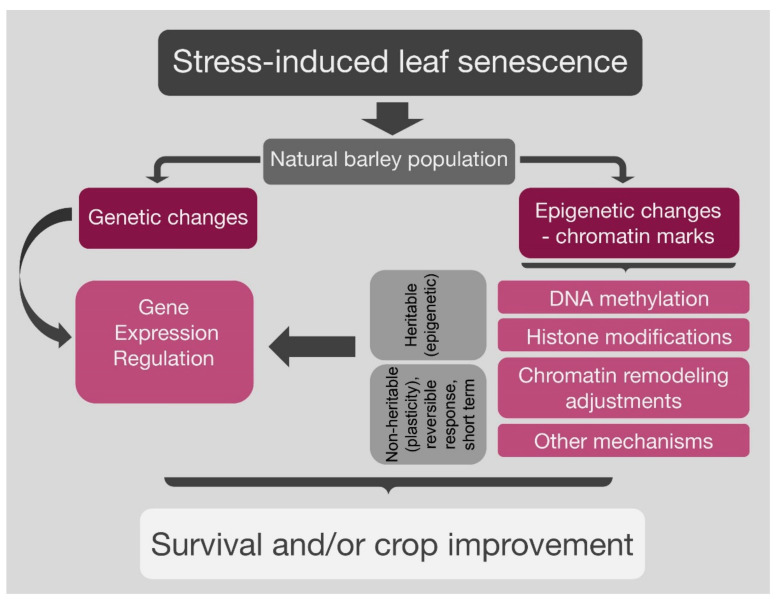
Gene expression regulation through genetic and epigenetic modifications in natural population in response to environmental stimuli. The genetic and epigenetic changes may act independently or interdependently in regulating the gene expression, which may result in heritable (stress memory) changes and lead to survival and/or crop improvement.

In conclusion, crop senescence involves major reprogramming of gene expression, and recent research has revealed complex regulatory mechanisms, including the hierarchical action of many TFs, but also a higher-order regulation via alterations in chromatin structure. Epigenetic control of the level of senescence-inducing signals, which is connected to the overall environmental cues and the developmental program, is then possible. There is rapidly accumulating evidence that gene regulation of these pathways includes differential changes in chromatin status, switching from transcriptionally inactive heterochromatin to actively transcribed euchromatin, and vice versa. This control level is implemented by different but interacting and often interdependent epigenetic mechanisms, including, described above, DNA methylation, covalent histone modifications, and non-covalent chromatin remodeling. It may allow a rapid response to signaling and stimuli when the expression of a group of proteins (which can range from tens to thousands) needs to be adjusted immediately. The genetic and epigenetic changes may act alone or together and regulate the gene expression, which may result in nonheritable and heritable (stress memory) changes and may lead to survival and/or crop improvement (Figure 1).

The barley crop model for early and late events during dark-induced leaf senescence [11], which also determines the critical time limit for reversal of the senescence process, has shown the most evident differences in gene medleys between DILS and developmental senescence that included inter alia DNA and RNA modifications active in DILS. This suggests the possibility of a yet-to-be-discovered additional switch between cell survival and cell death. Using large-scale *Arabidopsis* expression data reported by Breeze et al. [89] and available barley leaf senescence-related microarrays [11,90,91], we determined the transcription pattern during senescence of genes which are either known or suggested to be involved in plant DNA methylation processes. Genes such as AGO10 (which encodes a member of the elongation initiation factor and plays a central role in RNA silencing processes, as essential components of the RNA-induced silencing complex), MET1 and ROS1 were significantly regulated at the transcription level in DILS. In developmental leaf senescence opposite to DILS MET1 and ROS1 transcript levels decreased before the leaves were fully expanded, but e.g., in both DILS and DLS histone acetyltransferase HAC1 is highly expressed.

The conclusions from the research contributed to the formulation of the hypothesis that chromatin-remodeling mechanisms in response to induced senescence as environmental stimuli control the rate of leaf senescence by: (i) initiation of leaf senescence, (ii) control of senescence-dependent remobilization, (iii) leaf passage from senescence to the death phase.

It is desirable to design new breeding strategies in which the epigenetic variability should be taken into consideration (so called epibreeding). Epigenetic variants in crops might represent an additional and timely resource for crop breeding. Epigenetic variants with potential agronomic interest that have been identified in several crop species are dwarf phenotypes in rice [92], anthocyanin production in apple [93], increased seed protein content and decreased oil content in oilseed rape [94], sex determination in melon [95] and fruit ripening in tomato [96]. Epigenetic variants of agronomic interest can also be obtained using chemical treatments such as exposure to the methylation inhibitor 5-azacytidine, e.g., early flowering in strawberry [97]. Natural environmental stressors can also be used as a source of epigenetic variation for physiological traits in crops, e.g., drought and salt tolerance in rice [98]. Epibreeding does not require selection methods that would differ in nature from conventional crop breeding methods. Conventional approaches based on genetic variation (e.g., cross-breeding followed by selection) can be transferred to epibreeding with obvious differences in terms of epigenetic variant induction, production, and propagation. The result of classical crop breeding methods applied to standing, environmentally or artificially induced, epigenetic variation, might be stabilized when necessary by using vegetative propagation [99]. Selection trials might be assisted by using molecular epigenetic markers, as is already done with genetic markers. Epigenomic marks associated with phenotypic variants of interest have already been identified. Differentially methylated regions (DMRs) were used for the early detection of hypomethylated genes associated with the unwanted oil palm fruit “mantled” abnormal phenotype (catastrophic homeotic transformation, parthenocarpy and marked loss of yield) [100].

It is thus necessary to deepen our investigation of the epigenetic regulators in crops during stress-induced senescence and their underlying molecular mechanism. Understanding the mechanism of epigenetic regulators and their regulatory networks in this process in crops will be a potential tool for further exploitation toward sustainable agriculture. It seems even more realistic with the advances in genomic technologies and the cost lowering of next-generation sequencing. Like MAS (marker-assisted selection), epigenetic marker-assisted selection could also be initiated.

## Figures and Tables

**Table 1:** Examples of loss-of-function and gain-of-function *Arabidopsis* mutants and transgenic lines showing senescence-affected phenotype.

*Arabidopsis* Mutant/Line Name	Gene Affected	Epigenetic Process Affected	Mutant Phenotype	Reference
anti-sense AtHD1 transgenic line	histone deacetylase 1 (AtHD1 or AtHDA19)	histone deacetylation	Reduced AtHD1 transcript level; pleiotropic developmental defects; early senescence.	[44]
HDA6 axe1-5; HDA6-RNAi	histone deacetylase HDA6	histone deacetylation	Higher H3 acetylation; delayed flowering and leaf senescence; down-regulation of SAGs (SAG12 and SEN4).	[45]
HDA9 mutant	histone deacetylase HDA9	histone deacetylation	Delayed leaf yellowing and senescence.	[46]
hls1 mutant	histone acetyl-transferase HOOKLESS1 (HLS1)	histone acetylation	Accelerated senescence; impaired response to ABA; impaired responses to pathogen infections.	[47]
hac1 mutant	HAC1—histone acetyltransferase	histone acetylation	Delayed age-related developmental senescence, but normal dark-induced senescence; Late flowering.	[48]
jmj16 mutant	JMJ16—H3K4 demethylase	histone demethylation	Increased H3K4me3 at WRKY53 and SAG201; early-senescence (ES) phenotype.	[49]
ref6-1 mutant	REF6—H3K27 demethylase	histone methylation	Late flowering; Delayed leaf senescence; H3K27me3 hypermethylation; decreased activity of key regulatory and functional genes of leaf senescence process.	[50]
SUVH2 overexpression plants	SUVH2 histone methyl-transferase	histone methylation	Inhibition of key regulators: SIRK, SAG101, ANAC083, SAG12, SAG24, related to WRKY53 function repression; increased H3K27me2 and H3K27me3 level; delayed leaf senescence.	[51]
SUVH2 overexpression plants	SUVH2 histone methyl-transferase	histone methylation	Limited activity of nearly half of the senescence-related regulatory factors (SRRFs); delayed leaf senescence.	[52]
atm mutant	ATM—transducer of double-strand breaks (DSBs) signal	histone methylation	Accumulation of DSBs; severe yellowing; premature senescence.	[53]
antisense MET1 gene	METHYLTRANS-FERASE 1 (MET1)	DNA methylation	Genome hypomethylation; inappropriate gene and transposon transcription; delayed senescence; developmental defects.	[54]
Drd1-6 mutant	DRD1	Chromatin remodeling	Overexpressed DRD1 gene; significant SAGs inhibition during dark-induced and natural senescence; delayed senescence.	[55]
ore7-1D mutant	ORE7	Chromatin remodeling	Overexpression of ORE7 gene; SAGs inhibition; delayed senescence.	[56]
ddm1-2 mutant	DDM1	Chromatin remodeling	SAGs inhibition during dark-induced and natural senescence; delayed senescence.	[55]
arf2 mutant	ARF2—auxin-dependent senescence regulator	Additional mechanisms	miR390-mediated production of TAS3—transcriptional silencing complex protein; delayed senescence.	[57]

## Abbreviations

ABA	Abscisic acid
ATM	Ataxia telangiectasia mutated (serine/threonine-protein kinase)
ChIP	Chromatin immunoprecipitation
ChIP-seq	Chromatin immunoprecipitation-sequencing
CMT3	Chromomethylase 3
DILS	Dark-induced leaf senescence
DNMT	DNA methyltransferase
DRD1	Chromatin remodeling protein 1
DSB	Double-strand break
H3K9ac	Histone H3 at lysine 9 acetylation
H3K4me2	Histone H3 at lysine 4 dimethylation
H3K4me3	Histone H3 at lysine 4 trimethylation
H3K9me2	Histone H3 at lysine 9 dimethylation
H3K27me3	Histone 3 lysine 27 trimethylation
HAT	Histone acetyltransferase
HDA6	Histone deacetylase 6
HDA9	Histone deacetylase 9
HDAC	Histone deacetylase
MET1	DNA methyltransferase 1
miRs	MicroRNAs
ORE7	AT-hook protein ORESARA 7
PCD	Programmed cell death
PPH	Pheophytin pheophorbide hydrolase
PRR9	Pseudo-response regulator 9
PTM	Post-translational modifications
RdDM	RNA-directed DNA methylation
RNAi	RNA interference
ROS1	Repressor of silencing 1
SAG	Senescence-associated gene
SAG12	Senescence-specific cysteine protease
SAG24	Senescence-associated protein
SAG101	Senescence-associated carboxylesterase 101
SEN4	Xyloglucan endotransglucosylase/hydrolase
SIRK	Senescence-induced receptor-like serine/threonine-protein kinase
SRRF	Senescence-related regulatory factor
SUVH2	SU(VAR)3–9 homolog 2 histone methyltransferase
TE	Transposable element
TF	Transcription factor
WRKY41	Probable WRKY transcription factor 41
WHIRLY1	Single-stranded DNA-binding protein WHY1
